# The Efficacy of Anti-vibration Gloves

**DOI:** 10.1007/s40857-015-0040-5

**Published:** 2016-02-03

**Authors:** Sue Hewitt, Ren Dong, Tom McDowell, Daniel Welcome

**Affiliations:** 1 Health and Safety Executive, Harpur Hill, Buxton, SK17 9JN UK; 2 Engineering & Control Technology Branch, Health Effects Laboratory Division, National Institute for Occupational Safety and Health, Morgantown, WV 26505 USA

**Keywords:** Anti-vibration gloves, Hand–arm vibration, Hand–arm vibration syndrome, Personal protective equipment

## Abstract

Anyone seeking to control the risks from vibration transmitted to the hands and arms may contemplate the use of anti-vibration gloves. To make an informed decision about any type of personal protective equipment, it is necessary to have performance data that allow the degree of protection to be estimated. The information provided with an anti-vibration glove may not be easy to understand without some background knowledge of how gloves are tested and does not provide any clear route for estimating likely protection. Some of the factors that influence the potential efficacy of an anti-vibration glove include how risks from hand–arm vibration exposure are assessed, how the standard test for a glove is carried out, the frequency range and direction of the vibration for which protection is sought, how much hand contact force or pressure is applied and the physical limitations due to glove material and construction. This paper reviews some of the background issues that are useful for potential purchasers of anti-vibration gloves. Ultimately, anti-vibration gloves cannot be relied on to provide sufficient and consistent protection to the wearer and before their use is contemplated all other available means of vibration control ought first to be implemented.

## Introduction

The connection between use of vibrating power tools and the associated health effects referred to as hand–arm vibration syndrome (HAVS) has been known for around a century. In the modern workplace, health effects associated with power tool use are still commonly reported and there are hundreds of new cases reported every year in the UK [1]. When attempting to manage exposure to hand–arm vibration in the workplace, and having exhausted all the other possible approaches to managing the problem, the question of personal protective equipment (PPE) will inevitably arise. Anti-vibration gloves are available, which are typically made from materials such as resilient gel, foam or rubber-like material or an array of air bladders. This paper considers the issues that surround the selection and use of anti-vibration gloves as PPE for hand–arm vibration.

In the UK, the Health and Safety Executive produced guidance in 2005 on the control of risks from hand–arm vibration [2]. Part six of the guidance contains technical information on anti-vibration gloves and explains the main points to be considered very succinctly. The guidance concludes that employers should not assume that anti-vibration gloves reduce vibration exposures unless specific data are available for the particular combination of glove and tool used. This paper provides further background and updated information; however, the guidance remains unchanged.

To understand the technical considerations relating to the prospective use of anti-vibration gloves, it is necessary to know how exposure to hand–arm vibration is assessed according to current international standards and how a vibration-reducing glove is tested before it can be marketed as an anti-vibration glove in Europe and USA.

## Assessment of Hand–Arm Vibration Exposure

**Fig. 1:**
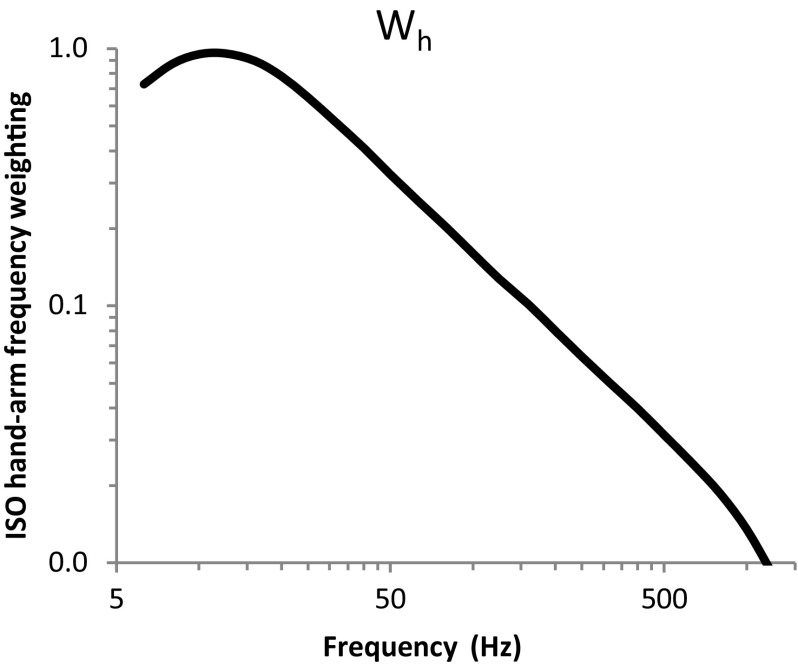
ISO 5349-1:2001 [3] hand–arm frequency weighting, $W_\mathrm{h}$

The international standard for measurement and assessment of exposure to hand–arm vibration is ISO 5349:2001, parts 1 and 2 [3, 4]. These standards define how the vibration to which an individual is exposed is measured and evaluated in terms of the frequency-weighted vibration total value at or near to the gripping zone. The hand–arm frequency weighting defined in ISO 5349-1:2001 is shown in Fig. 1.

The hand–arm frequency weighting gives most weight to low frequencies between 6.3 and 25 Hz. This is relatively low compared with most types of power tool, which typically have a main operating frequency in the range of 25–150 Hz [5].

**Fig. 2:**
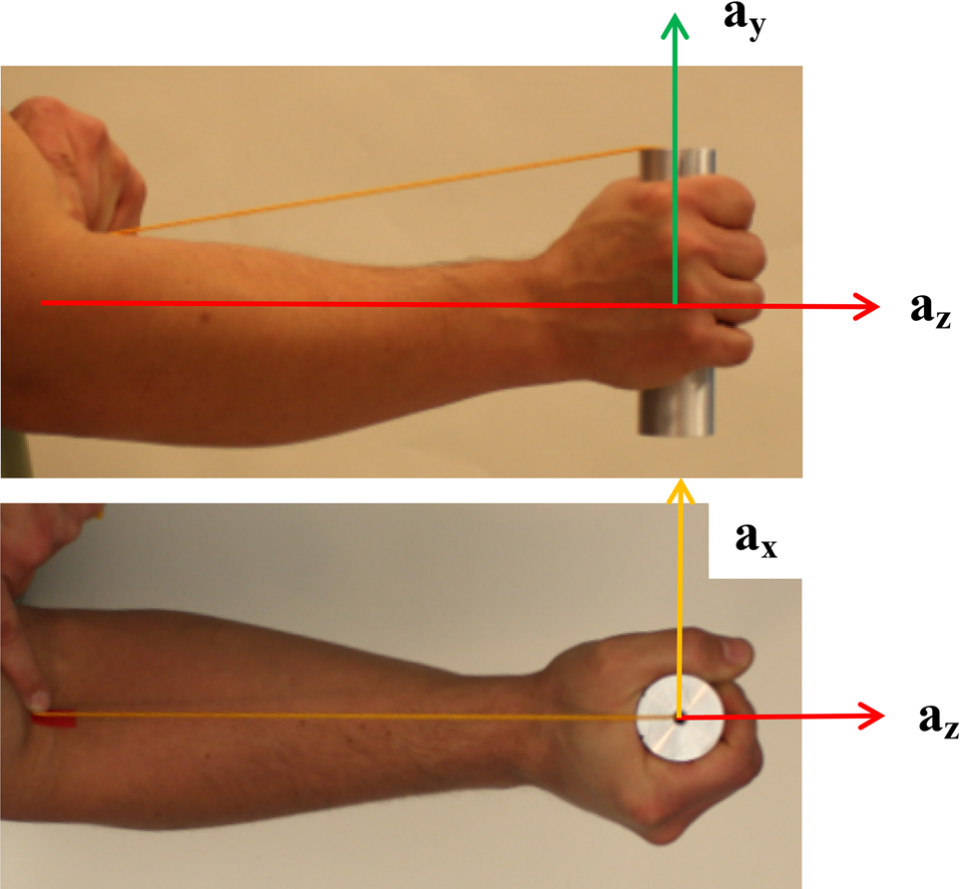
Illustration of $x,\,y$ and *z* axes used for testing according to the thenar region-based biodynamic coordinate system from Dong et al. [6]

The frequency-weighted vibration total value is a single figure relating to the vibration on the surface of the machine that the operator is in contact with. It combines the measures of the vibration in three orthogonal directions; axes: $x,\,y$ and *z*. Figure 2 shows the $x,\,y$ and *z* axes as described in a recent paper [6] as they relate to the axes used for assessment of the performance of an anti-vibration glove. The hand–arm frequency weighting is applied to the vibration in each of the three axes, before they are combined to give the vibration total value.

When assessing vibration exposure according to ISO 5349-1:2001, the vibration total value is combined with information on the duration of the exposure to vibration to give a daily vibration exposure, *A*(8),  expressed in units of m/s$^{2}.$

## Assessment of Anti-vibration Glove Performance

The current international standard that should be applied to a glove before it can be marketed as an anti-vibration glove is ISO 10819:2013 [7]. The test described in this standard involves applying a defined signal to a vibrating handle and then measuring how much of that vibration is transmitted through the glove to the palm of the wearer. To achieve this, the vibration is measured simultaneously on the surface of the handle and in the palm of the hand using an adaptor. This enables the vibration transmitted through the glove to be calculated. The test uses a band-limited random vibration signal. The vibration magnitude used for the test is defined in all the one-third octave frequency bands from 25 to 1600 Hz. This range is selected based on the possible effective frequency of anti-vibration gloves and the frequency range of concern for hand–arm vibration exposure defined in ISO 5349-1:2001. The values that are produced by application of the test are referred to as transmissibilities. The transmissibility values are calculated using hand–arm frequency-weighted vibration magnitudes to determine whether the glove reduces the vibration that is transmitted to the wearer. When the overall results of the glove transmissibility measurements are calculated, they are expressed as two values:

- $\bar{T}_{(\mathrm{M})},$ the average result from the 25 Hz one-third octave band to the 200 Hz one-third octave band and
- $\bar{T}_{(\mathrm{H})},$ the average result from the 200 Hz one-third octave band to the 1250 Hz one-third octave band.

A transmissibility of 1.0 means that all of the vibration is transmitted through the glove material to the wearer. If the transmissibility is less than 1.0, it indicates that the glove is reducing the amount of vibration that is being transmitted. If the transmissibility is more than 1.0, it indicates that the glove is amplifying the vibration.

**Fig. 3:**
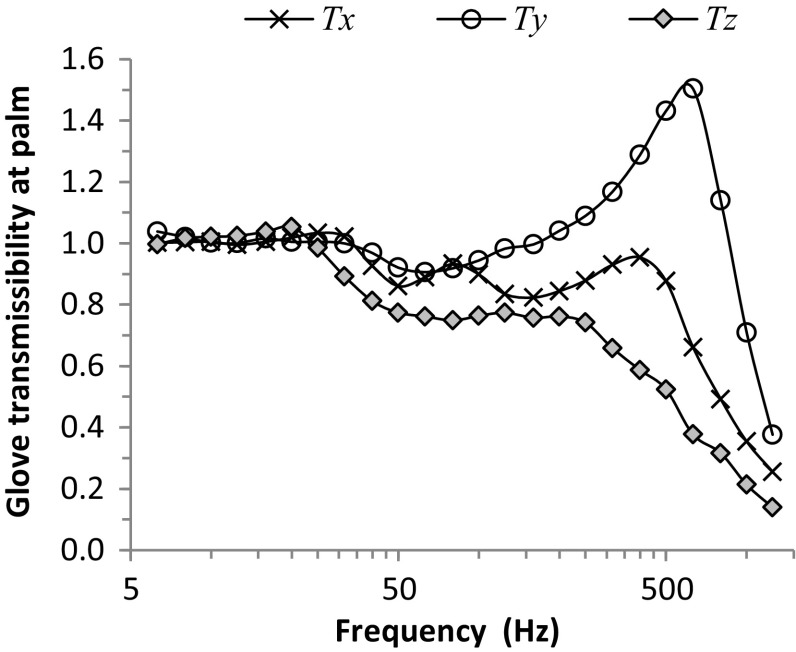
Example transmissibilities of an air bladder glove at the palm (from Dong and colleagues [9])

To be CE marked and marketed as an anti-vibration glove in Europe, a glove must first satisfy both the criteria for the transmissibility set in ISO 10819:2013:

$$\begin{aligned} \bar{T}_{(\mathrm{M})}\le & {} 0.90,\quad \mathrm{and}\\ \bar{T}_{(\mathrm{H})}\le & {} 0.60. \end{aligned}$$

These criteria mean that the glove must provide on average at least 10 % reduction in the frequency-weighted vibration between 25 and 200 Hz and must provide on average at least a 40 % reduction in the frequency-weighted vibration between 200 and 1250 Hz.

The ISO 10819:2013 standard also specifies the maximum thickness of the material in the palm of an anti-vibration glove and also at the fingers, although the transmissibility of the glove is only tested at the palm.

Gloves that satisfy the transmissibility criteria and also satisfy the requirements for thickness of the material can be given a CE mark and sold as an anti-vibration glove in the EU.

## Factors Affecting the Apparent Performance of Anti-vibration Gloves

### ISO 5349 Hand–Arm Frequency Weighting

Most anti-vibration gloves do not provide much reduction in vibration transmission at frequencies below 25 Hz at the palm of the hand and below 160 Hz at the fingers [8, 9]. A typical anti-vibration glove may in fact cause slight amplification of vibration at low frequencies [8, 9]. Figure 3 shows examples of the transmissibilities of an air bladder glove measured in the *x*-, *y*- and *z*-axes at the palm of the hand [10]. Figure 3 shows that the typical glove transmissibilities are close to 1.0 at low frequencies where the hand–arm frequency weighting is at its peak. The main reductions in transmissibility also depend on the vibration axis and the applied hand forces [10, 11]. The gloves can usually become more effective with the reduction of the applied hand forces [11] but certain hand forces are required to control a tool.

At more than 160 Hz, the ISO 5349-1:2001 hand–arm frequency weighting reduces the vibration signal to less than one tenth of its actual value. This, in combination with the fact that most machines have operating frequencies below 160 Hz, makes it difficult for any glove to provide very much reduction of the frequency-weighted vibration for the fingers; the frequency weighting limits the contribution made by the higher frequencies to the overall vibration magnitude. The ISO 5349-1:2001 hand–arm frequency weighting is currently under review [12], although it is unlikely to be changed in the near future.

The current hand–arm frequency weighting is a single weighting used to assess vibration exposure for all possible health effects that might be associated with hand–arm vibration, but the origins of the frequency weighting are not related to health. The underlying research was based on equal vibration sensation contours of the entire hand–arm system [13]. As there are many different health effects encompassed by the term HAVS, it is possible that the current hand–arm frequency weighting may be more appropriate for some of these health effects than for others. Evidence from studies of health effects and biodynamic modelling [14, 15] indicates that the current hand–arm weighting may be most suited to health effects in the palm–wrist–arm substructures. Further evidence relating to health effects at the fingers [16, 17] indicates that gloves may be more beneficial than is predicted by the limited reductions in frequency-weighted vibration exposure that gloves provide. Other studies of the neurological health effects of hand–arm vibration exposure [18, 19] also indicate that high frequencies may be more damaging. These findings point to the possibility that the frequency weighting is inadequate for assessing the risk of developing some of the health effects associated with hand–arm vibration exposure. The question of the contribution of higher frequencies to the development of health effects and the issues relating to the hand–arm frequency weighting are discussed in more detail by Hewitt et al. [20]. Ultimately however, because the exact mechanism or mechanisms of damage for vascular and neurological finger symptoms have not been clearly identified, and the exposure–response relationship for HAVS remains ill-defined [21], it is difficult to establish a suitable frequency weighting or weightings to predict the different health effects of vibration. In the absence of any strong evidence to support alternatives, the current frequency weighting is unlikely to be changed at any time in the near future [12] and consequently the technique for assessment of the performance anti-vibration gloves is also unlikely to be changed.

### Limitations of the Standardised Glove Test

#### Transmissibility of a Glove in Different Directions

**Fig. 4:**
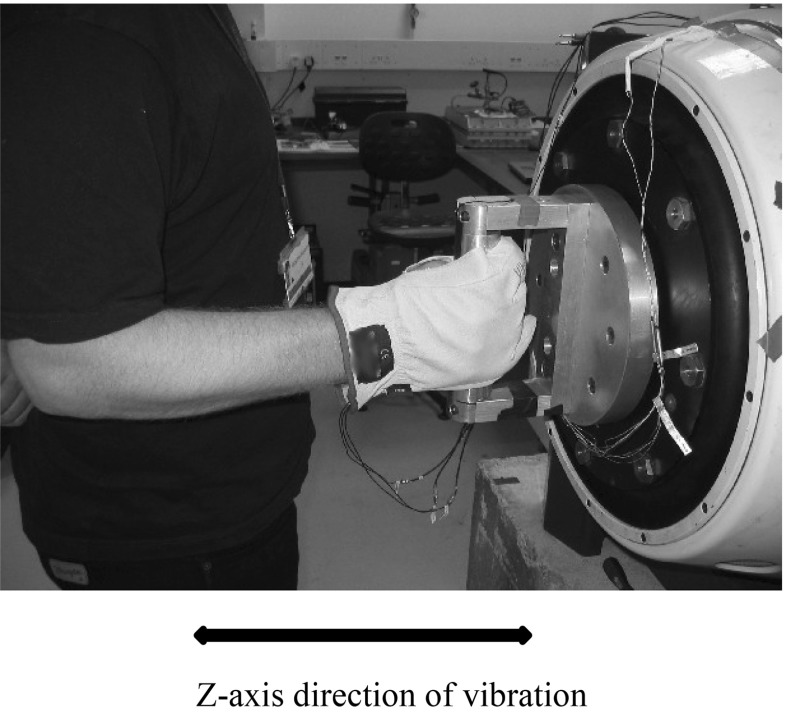
A glove test set-up, testing in compression in the *z*-axis

**Fig. 5:**
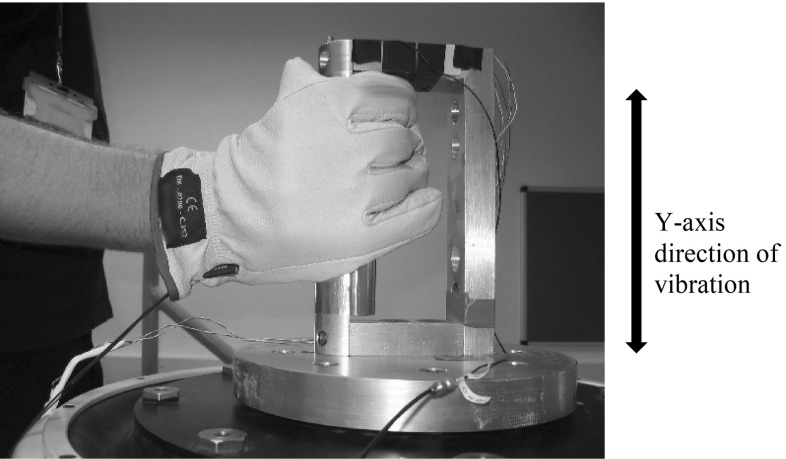
A glove test set-up, testing in shear in the *y*-axis

The frequency-weighted vibration total value is a combination of the vibration measured in the three orthogonal axes as shown in Fig. 2. ISO 10819:2013 only specifies measurement of the performance of a glove in the *z*-axis with the material acting in compression. An example of the test set-up for *z*-axis testing is shown in Fig. 4. The biodynamic models developed by Dong et al. [15] demonstrate how the transmission of vibration through the glove is affected by the physical characteristics of the individual components of the finger and the palm–wrist–arm structure. The transmissibility can be very different in shear, which is in a direction through the hand as represented by the *y*-axis in Fig. 2 and shown in Fig. 5.

**Fig. 6:**
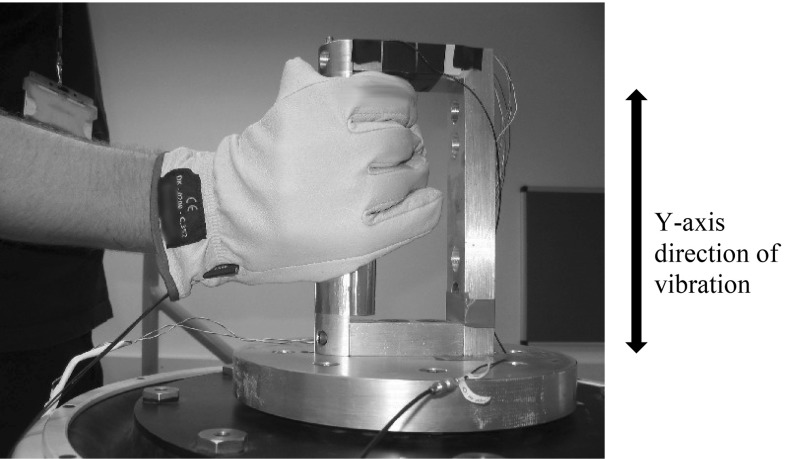
Example transmissibilities of an air bladder glove at the fingers (from Welcome and colleagues [23])

The examples of transmissibilities in the three axes, $T_{x},\, T_{y}$ and $T_{z}$ measured at the palm of the hand for an air bladder glove are shown in Fig. 3. The differences at higher frequencies are not only due in part to the difference in the properties and behaviour of the glove material in shear, but are also due to the differences in the biodynamics of the hand–arm system. It is clear then that the transmissibility of an anti-vibration glove is both direction and posture specific. Because the effective mass at the palm is usually the highest along the *z*-axis, the vibration reduction of a glove is usually most effective in this direction, but because the standard glove test is only applied in this direction, the results will usually overestimate the total effectiveness of the glove.

#### Transmissibility of a Glove at the Fingers

The vibration transmissibility of a glove at the fingers has been shown to be much different from the transmissibility at the palm of the hand [22]. ISO 10819:2013 sets requirements for distribution and thickness of the vibration-reducing material at the fingers, for compliance with the standard test criteria. These requirements do not actually increase the glove effectiveness, but they may make production of gloves more difficult [22]. Furthermore, the actual performance of a glove is only measured at the palm.

Figure 6 shows an example of air bladder glove transmissibilities measured at the fingers in the *y*-axis and in the combined *x*- and *z*-axes. The transmissibilities for the *x*- and *z*-axes are combined because it is difficult to reliably separate the two directions when considering the fingers [22]. The vibration transmissibility of a glove at the fingers has been shown to be generally much higher than at the palm of the hand, meaning that gloves are much less effective at reducing vibration transmitted to the fingers than to the palm of the wearer [23]. This is mainly due to differences in the apparent mass of the fingers compared with the rest of the hand–arm system [23]. Estimates of the protection afforded to the fingers have shown that anti-vibration gloves would actually have little value for reducing finger-transmitted weighted vibration, except in some special cases [20].

### Vibration Spectrum from the Tool

The performance of an anti-vibration glove will depend on the acceleration spectrum and direction on the power tool handle. A transfer function method has been used to estimate the vibration reduction potential of a glove at the palm and at the fingers for a variety of different power tool spectra [10, 24]. The data show that for the vibration transmissibility at the fingers, any reduction in vibration is not significant for most tools.

When the transmissibility at the palm is considered, the performance of the glove is expected to depend heavily on the main operating frequency of the tool [10]. For tools that work at low frequencies, such as sand rammers, very little vibration reduction is predicted. For tools operating at medium frequencies, around 30–50 Hz, such as chipping hammers, the predicted reductions are typically between 5 and 20 %. In one example for an impact drill, which has a large amount of high frequency content in the vibration spectrum for each axis, the glove is predicted to reduce the vibration by more than 30 %. These data are similar to results from an earlier study [8] which showed the estimated range of performance for one type of gel and foam glove was from 3 % amplification when applied to the vibration spectra from an angle grinder with a main operating frequency of 100 Hz, to 30 % reduction for a multi-use sanding tool with a main operating frequency of 315 Hz. These estimates are, however, theoretical and have not been corroborated by measurement. In practice, a valid measurement of the transmissibility of a glove on a real tool is very difficult to achieve [8] due to the influence of factors such as the mounting of the transducers as well as due to changes in grip and push forces affecting measured vibration magnitudes.

### Design Limitations of Anti-vibration Gloves

The effectiveness of an anti-vibration glove depends on both the material properties of the glove and the effective mass of the hand–arm system [23]. While the glove materials can vary substantially, the natural dynamic properties of the hand–arm system cannot be substantially changed. This is one of the major factors that limit the effectiveness of the anti-vibration gloves.

The design of anti-vibration gloves is also limited by the need for gloves to be wearable and safe. Thicker, softer materials will be more effective at reducing transmissibility, but increasing softness introduces issues with safe operation and adequate control. Thicker gloves may also result in the need for increased grip force to hold and control the tool, which may also potentially result in operator fatigue [25].

### Influences of Varying Forces and Individuals

The influence of applied forces is known to affect the amount of vibration transmitted through a glove [11, 26]. When a power tool is used for a real work task, the grip and push forces and working postures may be highly variable across a wide range of forces. The measurements of transmissibilities of anti-vibration gloves according to ISO 10819:2013 are carried out under controlled laboratory conditions. This includes controlling the amount of grip and push force applied during any measurement made as well as the posture adopted. In the standard test conditions in ISO 10819:2013, measurements of transmissibility at the palm are made using a grip force controlled to 30 N and the push force controlled to 50 N. How applicable transmissibility data measured at one specific level of force might actually be for the real work situation has not been well established, but grip and push forces are bound to vary considerably in real work situations.

The overall transmissibility of a glove at the palm of the hand has been shown to vary by as much as 20 % from individual to individual even under controlled laboratory test conditions [27]. When transmissibilities are measured, they are typically averaged across operators and this limits their applicability to the general population depending on the number and physical characteristics of those used as test subjects. The number of test subjects required for the ISO 10819:2013 test has been increased from 3 to 5 to take this into account, but even this may not be sufficient in some cases [20]. It is difficult to reach a consensus for further increasing the number of subjects, as the increase will largely increase the cost of the test.

### Inter-relationship Between Influencing Factors and Prediction of Performance

Many factors such as the vibration spectrum of a power tool, the main direction of the vibration, the transmissibility of the glove in that direction, the physical characteristics of the wearer and the posture and amount of force applied by the wearer to the vibrating surface will all be combined to define a level of transmissibility which is specific to that set of circumstances. The tool vibration spectrum and biodynamic responses themselves are also influenced by the hand forces, vibration direction, operating styles, working materials and individuals. Therefore, the factors influencing the assessment, performance and effectiveness of an anti-vibration glove are interrelated and their interactions may be complex. The large number of influencing factors and their interactions make it very difficult to accurately predict or measure the actual individual performance of a glove. They also make anti-vibration gloves unreliable as a form of PPE.

## Summary and Conclusions

There are many factors that influence the measured transmissibility of an anti-vibration glove and the potential that a glove has to provide protection to the wearer. These factors include the effect of different directions and different frequencies of vibration and how they interact, the differences in transmissibility between the palm and the finger, and the variations due to different forces applied to the glove and due to different physical characteristics of the wearers. Anti-vibration gloves can reduce vibration components at very high frequencies $({\ge }500\,\mathrm{Hz}),$ especially when a low hand coupling force is applied. However, the hand–arm frequency weighting defined in ISO 5349-1:2001 required to evaluate the exposure for risk assessment restricts the measured efficacy of an anti-vibration glove.

Other ways of controlling vibration exposure, such as eliminating the need for the exposure, using low-vibration machinery and minimising exposure times are far more likely to be effective and ought first to be adopted. Thicker gloves are more likely to be effective at reducing vibration transmission, but may increase the grip forces needed to safely operate the machine and reduce manual dexterity, so the pros and cons of anti-vibration gloves ought to be carefully balanced if their use is to be considered.
